# What impact do prescription drug charges have on efficiency and equity? Evidence from high-income countries

**DOI:** 10.1186/1475-9276-7-12

**Published:** 2008-05-02

**Authors:** Marin C Gemmill, Sarah Thomson, Elias Mossialos

**Affiliations:** 1 LSE Health, London School of Economics and Political Science, Houghton Street, London, WC2A 2AE, UK

## Abstract

As pharmaceutical expenditure continues to rise, third-party payers in most high-income countries have increasingly shifted the burden of payment for prescription drugs to patients. A large body of literature has examined the relationship between prescription charges and outcomes such as expenditure, use, and health, but few reviews explicitly link cost sharing for prescription drugs to efficiency and equity. This article reviews 173 studies from 15 high-income countries and discusses their implications for important issues sometimes ignored in the literature; in particular, the extent to which prescription charges contain health care costs and enhance efficiency without lowering equity of access to care.

## 1. Background

The notion that user charges improve efficiency is regarded by some as self-evident. Not only do user charges reduce the welfare loss caused by full insurance, but they also help to contain health care costs, encourage patients to choose more cost-effective forms of care, and are a valuable source of revenue for the health system. Yet there is growing evidence to suggest that the reverse might be true. Although user charges consistently lower health care use and, if carefully designed, can steer patients towards cost-effective care, they do not lead to long-term control of pharmaceutical spending and seem unlikely to contain total expenditure on health (not least because they can threaten patients' health). In spite of research suggesting that user charges are unlikely to contribute to health policy goals such as efficiency and equity, all OECD countries charge patients for some health services, most commonly for prescription drugs. The universal application of prescription drug charges in OECD countries may reflect anxiety about the rapid growth of pharmaceutical budgets [1], although many of these countries applied prescription drug charges before rising drug budgets became a pressing policy matter. Table 1 gives details of different forms of prescription drug charges.

**Table 1:** Direct and indirect forms of prescription drug charges and their incentives

**Form**	**Definition**	**Patient incentives**
**Direct**		
Co-payment	The user pays a fixed fee (flat rate) per item or service.	The patient may decrease the volume of drugs consumed or may decrease the number of prescriptions filled while increasing the size of each prescription. The patient has no incentive to consume cheaper drugs unless co-payments are lower for these drugs.
Co-insurance	The user pays a fixed proportion of the total cost, with the insurer paying the remaining proportion.	The patient may decrease the volume of drugs consumed and may only request a larger pack size if this produces savings. The patient has an incentive to consume cheaper therapeutic medications.
Deductible	The user bears a fixed quantity of the costs, with any excess borne by the insurer; deductibles can apply to specific cases or to a period of time.	When patients are not close to the deductible level, they may decrease the volume of drugs consumed and/or switch to cheaper therapeutic alternatives. As they near the deductible limit, they have an incentive to consume more drugs and more expensive drugs to push themselves over the deductible.
**Indirect**		
Reference pricing (RP)	A reference price refers to the maximum price for a group of equal or similar drugs that the insurer will reimburse the user. If the user chooses a drug that costs more than the reference price, he or she must pay the difference.	The patient is likely to decrease his or her consumption of drugs that are priced above the reference price and switch to alternative drugs priced at or below the reference price.
**Differential charges**		
Multi-tier formularies	Typically, these contain two or three tiers. The first tier consists of generic drugs, which have the lowest co-payment. The second and third tiers generally comprise brand-name drugs, which can be split into preferred and non-preferred drugs (where non-preferred drugs are the most expensive in the tier). Multi-tier formularies are most commonly used in the United States.	The patient has an incentive to switch from brand-name medications to generic medications and from non-preferred medications to preferred medications.

This article reviews the literature on user charges for prescription drugs in high-income OECD countries with a view to assessing their impact on efficiency and equity. The substantial body of literature on prescription drug charges already includes several reviews. However, the remit of most of these reviews is constrained by a focus on, for example, specific populations [2-4]; a sub-set of the literature such as studies from the United States, the United Kingdom, and/or Canada [5-9]; specific forms of prescription drug charges such as reference pricing [10,11] and tiered formularies [12]; or the main articles in the area [7,13-16]. We add to existing reviews by covering studies carried out in a wider range of high-income countries and reviewing papers published in languages other than English. We also go beyond them in attempting to assess the relationship between prescription drug charges, efficiency, and equity. The article begins with a brief overview of economic and policy arguments in favour of user charges. It then describes the methods we used to identify relevant literature and notes some of the limitations of our approach. After reviewing the literature, we conclude with a discussion of policy implications.

## 2. How can user charges improve efficiency?

Before reviewing the literature, we outline some key economic and policy arguments in favour of user charges as a means of improving efficiency. Understanding these arguments may help to explain why user charges continue to be advocated, even when there is substantial evidence of their potentially detrimental effect on both efficiency and equity. We also define what we mean by efficiency and equity.

### Willingness to pay and welfare loss

Economic arguments in favour of user charges are based on the concept of allocative efficiency, which deems resources to be efficiently allocated when people are willing to pay for a commodity at a price that reflects the marginal cost of producing the commodity [17]. This has two ramifications. First, only those who are willing to pay should have access to a particular commodity. Second, providing a harmful or ineffective commodity to those willing to pay for it is efficient, whereas providing an effective and beneficial commodity to those unable to pay for it is inefficient. For example, if the presence of health insurance means that health care is free at the point of use, the consumption of health care will not reflect the marginal costs of its production, leading to welfare loss since scarce resources might be better spent on producing and consuming other commodities [18]. User charges redress this loss by reinstating price: those willing to pay the price may use health care, those unable to pay must do without. From an economic perspective, any reduction in the use of health care following the introduction of user charges contributes to allocative efficiency, regardless of the distributional or health consequences.

We might ask what relevance allocative efficiency has for policy making in health care. If it is to be understood as a normative concept, then we must assume either that the distributional and health consequences are of no importance or, if they are important, that all individuals in a given society share the same level of income, the same tastes and preferences, and the same risk of ill health, etc. [17,19]. But neither assumption reflects reality. Policy makers in all OECD countries show demonstrable concern for population health and equity of access to health care, albeit to varying degrees, while the people living in these countries experience different levels of income and health. We therefore prefer to use a definition of efficiency that explicitly refers to the external criterion of health improvement. Under this definition, an efficient allocation of health care resources would be one that maximises health gain, where health gain is measured in a standardised manner (for example, through quality-adjusted life years) [20]. For equity, we consider two dimensions. Equity in finance requires richer people to pay more for health care, as a proportion of their income, than poorer people [21]. Equity of access to health care implies access to health care based on need rather than ability to pay. Because equity of access is difficult to measure, most studies employ equal use of health care as a proxy for equal access, as we do in our concluding discussion.

### Containing health care costs

Economic theory would consider any reduction in use attributed to user charges as an improvement in allocative efficiency, regardless of the value or effectiveness of the health services foregone. However, many non-economists assume that, faced with user charges, rational consumers will forego the health services of least benefit to them (certainly those that are potentially harmful and perhaps those that are less effective). In this way, they argue, reduced use will not adversely affect health, but will help to contain costs and make health care more effective. Does this assumption hold? If patients do not have sufficient information to make rational choices, they may forego or delay useful treatment, perhaps damaging their health and leading to greater expenditure at a later date. Conversely, patients may turn to free (but more resource-intensive) forms of health care to avoid paying charges. The result might be higher rather than lower health care costs.

### Improving efficiency by raising revenue

User charges can raise revenue for the health system if they are set low enough not to deter significant amounts of use. This policy argument is more prevalent in low-income countries, where public resources for health care may be severely limited or non-existent. Under such circumstances, drawing on private resources to ensure an adequate supply of drugs, for example, could lead to health improvement, particularly if poorer people are exempt from user charges. In high-income countries it is hard to see how private finance could be more efficient (in contributing to health improvement) than public finance. Unless user charges exempt high users of health care, they are really a form of tax on people in poor health.

### Concern for efficiency or concern for third party payer budgets?

Which of these arguments in favour of user charges seems most convincing in the case of prescription drug charges? Applying the allocative efficiency argument to a form of health care that requires a doctor's prescription only underlines its irrelevance to health policy. The focus on patient use seems misplaced when it is doctors who make the decision to prescribe drugs. If charges are not applied across all health services, the use of substitutes for prescription drugs (often emergency care) may increase costs. This leaves the revenue-raising argument, which, as we have noted, is barely plausible in high-income countries. Is it possible, then, that the real reason third party payers impose prescription drug charges is to contain their own budgets by shifting costs to patients? And is this why so many countries exempt particular groups of people from paying prescription drug charges, in the hope that cost shifting will not adversely affect health? By studying policy outcomes, we may be able to provide an indirect answer to these and other questions.

## 3. Methods and limitations

We used existing literature reviews as the basis for our search, electronically tracing them forward in time by looking for studies that cited the articles we collected. We also searched the Internet and databases such as PubMed, EconLit, Blackwell's Synergy and Ingenta using combinations of the keywords shown in Table 2. In-country experts helped to identify some of the papers in languages other than English, which were then translated by colleagues. To enhance comparability we limited our search to articles focusing on high-income OECD members (Australia, Austria, Belgium, Canada, Denmark, Finland, France, Germany, Greece, Iceland, Ireland, Italy, Japan, Republic of Korea, Luxembourg, the Netherlands, New Zealand, Norway, Portugal, Spain, Sweden, Switzerland, the United Kingdom and the United States). We included any study that assessed the impact of any form of cost sharing for prescription drugs, including reference pricing, as well as studies that analyzed the impact of insurance coverage on prescription drug use. We did not include review articles or articles published after 2006.

**Table 2:** Keywords used to search for literature

**Main keyword**	**Combined with these keywords**
cost sharing	health
user charges	prescription drugs
user fees	medical care
co-payments	medical services
co-insurance	utilization
deductibles	access
reference pricing	compliance
insurance	adherence
insurance coverage	
reimbursement	

The review covers 173 articles (from 15 countries), 17 of which are in languages other than English. The most common country studied is the United States (US) and the most commonly-used US datasets are the Medicare Current Beneficiary Survey, the National Medical Expenditure Survey, the RAND Health Insurance Experiment, Medicaid claims data, and other administrative claims datasets. Most non-US studies also use data from administrative claims or national surveys.

We use tables to summarise our main findings and cite references, using the text for more detailed discussion. As a measure of quality, the tables specify the type of study carried out, the type of data analyzed, and the techniques used for analysis. Some studies are experimental (ES), some are based on a natural experiment (NS), and others are observational (OS). Data analysis is cross-sectional (CD), time-series (TD), or panel (time-series, cross-sectional) (PD). As most researchers used large datasets, we do not include information on sample size. The majority of studies used regression techniques to analyze data (R), but some reported descriptive statistics alone (NR). We do not go beyond this in assessing the quality of the research we review, mainly because efforts to determine the most appropriate method of analysis for each study depend on the study's objectives and sample characteristics.

A number of limitations are worth highlighting. No dataset is perfect. Analyses based on cross-sectional data may suffer from omitted variable bias (failure to account for explanatory variables), which can lead to biased coefficients and standard errors in the regressions [22]. The statistical methods used can also affect the quality of the results. For example, endogeneity may be an issue if individuals who are more likely to purchase insurance are also more likely to increase their consumption of prescription drugs once they have insurance, leading to biased and inconsistent estimates as well as invalid statistical tests [22]. Sample selection may be an issue where the dependent variable is only observed for a restricted, non-random sample. Regression estimates that do not account for sample selection will be biased because the researcher is unable to determine whether non-consumption is due to an individual not needing a prescription or due to an individual choosing not to purchase a prescription. Finally, few studies are able to determine whether dosage size changes in response to a price change, while few datasets give researchers insight into how doctors ease a patient's out-of-pocket burden or how cost sharing affects adherence to treatment.

## 4. The literature on prescription drug charges

We ask a range of policy questions relating to expenditure, use, and health to structure our review and to pave the way for discussion of the impact of prescription drug charges on efficiency and equity. First, we ask whether prescription drug charges affect expenditure on prescription drugs. If there is no effect on total prescription drug expenditure, then we conclude that prescription drug charges result in cost shifting from publicly or privately pooled pre-payment to patient payment at the point of use. This has clear implications for equity in finance and equity of access to care. Second, where there is some reduction in total expenditure on prescription drugs, we ask whether this is matched by any reduction in total health care expenditure or partially or wholly offset by increases in other health care costs. Third, we investigate whether reductions in prescription drug expenditure are caused by reductions in price or quantity. In the case of price, we ask whether the prescription drug charges were designed to encourage patients to choose lower-cost alternatives (for example, through reference pricing or tiered co-payments). In the case of quantity, we ask which patients are most likely to forego drugs and which drugs are most likely to be foregone. Finally, we consider the likely impact of prescription drug charges on health.

### 4.1. How do prescription drug charges affect expenditure on prescription drugs?

In this section we consider two types of expenditure: total prescription drug expenditure and patients' out-of-pocket expenditure. If patients are not particularly sensitive to changes in the price of prescription drugs, introducing or increasing user charges will have little effect on total prescription drug expenditure but will increase out-of-pocket spending on prescription drugs. Conversely, if patients are sensitive to changes in price, the impact on total expenditure will be greater, while the impact on out-of-pocket expenditure may be smaller as patients lower their use of prescription drugs.

Sixty-three papers examined the impact of cost sharing on total or out-of-pocket prescription drug expenditure using aggregate and non-aggregate data (see Tables 3 and 4). Aggregate data is defined as data collected at the macro-economic level so that individual- or household-specific information is not identifiable. The co-payment levels studied ranged from $0.50 to $35; co-insurance rates ranged from 0% to 95%. Most studies found that higher cost sharing lowered total prescription drug expenditure. Expenditure reductions ranged from a non-significant 0.04% of total prescription drug expenditure when moving from a two-tier to a three-tier formulary [23] to 58% of expenditure on ACE inhibitors when moving from a one-tier to a two-tier formulary [24]. Variation in the magnitude of expenditure reductions was influenced by contextual factors such as the size of the increase in user charges, the type of drugs associated with user charges, and the population groups subject to user charges. Some studies calculated expenditure elasticities to measure the extent of the change in total prescription drug expenditure in response to changes in the level of cost sharing. Most expenditure elasticity estimates ranged from -0.29 to -0.06 (that is, a 10% increase in cost sharing would result in a 0.6% to 2.9% decrease in total expenditure). The largest expenditure elasticity estimate (-1.07), a clear outlier, was from a study that focused on older people in the United States without any form of protection from user charges (for example, through employer-sponsored additional coverage or Medicaid, the publicly financed health insurance program for the poor), which may explain why this group was relatively sensitive to price [25]. Studies examining the impact of reference pricing found that it lowered prescription drug expenditure in the short term (generally in the first one or two years), but had little effect beyond this period.

**Table 3:** The impact of prescription drug charges on total prescription drug expenditure

**Variable**	**Expenditures**	**Studies**
Co-payment	-	**Hanau and Rizzi **[87] (IT, NS, TD, R); **Joyce et al. **[88] (US, OS, CD, R); **Lurk et al. **[27] (US, NS, CD, R); **Meissner et al. **[66] (US, OS, CD, NR); **Reeder and Nelson **[61] (US, NS, TD, R); **Smith **[89] (US, OS, CD, R)
Multi-tier formulary (vs. 1- or 2-tiers)	-	**Fairman et al. **[23] (US, NS, CD, R); **Gibson et al. **[90] (US, NS, PD, R); **Huskamp et al. **[24] (US, NS, CD, R); **Kamal-Bahl and Briesacher **[91] (US, OS, CD, R); **Motheral and Fairman **[28] (US, NS, CD, R); **Motheral and Henderson **[63] (US, NS, CD, R); **Nair et al. **[92] (US, NS, TD, R); **Thomas et al. **[93] (US, OS, CD, NR)
Co-insurance	-	**Alignon and Grignon **[94] (FR, OS, CD, NR); **Almarsdóttir et al. **[95] (IC, NS, TD, R); **Klaukka et al. **[96] (FI, NS, CD, R); **Liebowitz et al. **[48] (US, ES, CD, R); **Newhouse **[49] (US, ES, CD, R)
Deductible	-	**Van Vliet **[97] (NE, OS, CD, R); **Van Vliet **[98] (NE, OS, CD, R)
Mixed system	-	**Gaynor et al. **[99] (US, OS, PD, R); **Hong and Shepherd **[46] (US, OS, CD, NR); **Klick and Stratmann **[25] (US, OS, CD, R); **Smart and Stabile **[100] (CA, NS, CD, R); **Thomas et al. **[93] (US, OS, CD, NR)
Mixed system	0	**Grootendorst **[26] (CA, NS, PD, R)
Reference pricing (short-term effect)	-	**Anderson et al. **[59] (SW, NS, TD, R); **Grootendorst et al. **[64] (CA, NS, TD, R); **Grootendorst et al. **[101] (CA, NS, TD, R); **Mabasa and Ma **[39] (CA, NS, CS, NR); **Marshall et al. **[42] (CA, NS, TD, R); **Narine et al. **[44] (CA, NS, TD, NR); **Puig-Junoy **[102] (SP, NS, TD, R); **Schneeweiss et al. **[103] (CA, NS, TD, NR)
Reference pricing (short-term effect)	0	**Ulrich and Wille **[104] (GE, NS, TD, NR)
Reference pricing (long-term effect)	0	**Grootendorst and Stewart **[105] (CA, NS, TD, R); **Marshall et al. **[42] (CA, NS, TD, R); **Schneeweiss et al. **[106] (CA, NS, TD, R)
Change from		
co-payment to co-insurance	-	**Contayannis et al. **[107] (CA, NS, CD, R)
co-insurance to deductible and co-insurance	-	**Contayannis et al. **[107] (CA, NS, CD, R)
Insurance coverage		
Primary (vs. none)	+	**Artz et al. **[108] (US, OS, CD, R); **Danzon and Pauly **[109] (US, OS, CD, NR); **Gianfrancesco et al. **[110] (US, NS, CD, NR); **Smith and Garner **[111] (US, NS, CD, NR)
Supplementary (vs. none)	+	**Davis et al. **[112] (US, OS, CD, NR); **Dourgnon and Semet **[113] (FR, OS, CD, R); **Federman et al. **[114] (US, OS, CD, R); **Lillard et al. **[115] (US, OS, CD, R); **Long **[116] (US, OS, CD, R); **Poisal and Murray **[117] (US, OS, CD, NR); **Raynaud **[118] (FR, OS, CD, R); **Stuart et al. **[119] (US, OS, CD, R); **Weeks **[120] (US, NS, CD, NR)
Supplementary (vs. none)	+	**Yang et al. **[71] (US, OS, CD, R)
Supplementary (vs. none)	0	**Grignon and Perronin **[121] (FR, NS, CD, R)
Public supplementary (vs. private)	+	**Raynaud **[118] (FR, OS, CD, R)
Prescription limit	-	**Soumerai et al. **[37] (US, NS, TD, R)

Country: CA = Canada; FI = Finland; FR = France; GE = Germany; IC = Iceland; IT = Italy; NE = The Netherlands; MC = multiple countries; SP = Spain; SW = Sweden; US = United States

Type of study: ES = experimental study; NS = natural study; OS = observational study

Type of data analyzed: CD = cross-sectional data; TD = time-series data; PD = panel data

Type of statistical analysis used: R = regression techniques; NR = no regression techniques

**Table 4:** Estimates of the expenditure elasticity of demand for prescription drugs

**Paper**	**Type of cost sharing**	**Expenditure elasticity**
**Contayannis et al. **[107] (CA, NS, CD, R)	Change from co-payment to co-insurance	-0.16 to -0.12
**Klick and Stratmann **[25] (US, OS, CD, R)	Mixed system	-1.07
**Phelps and Newhouse **[122] (CA/UK, OS, CD, NR)	Co-insurance	-0.07^a^
**Smart and Stabile **[100] (CA, NS, CD, R)	Mixed system	-0.29 to -0.28
**Van Vliet **[97] (NE, OS, CD, R)	Deductible	-0.06
**Van Vliet **[98] (NE, OS, CD, R)	Deductible	-0.08

^a^unadjusted elasticity estimate (no regression used)

Country: CA = Canada; NE = The Netherlands; UK = United Kingdom; US = United States

Type of study: NS = natural study; OS = observational study

Type of data analyzed: CD = cross-sectional data

In general, the literature also found that having any form of insurance coverage (as opposed to none) increased total prescription drug expenditure. However, a Canadian study found that the provision of free prescription drugs for individuals aged 65 and over did not increase total prescription drug expenditure [26]. This result may be related to the fact that the author was only able to control for the unhealthiest respondents in the first year of the sample. Alternatively, additional insurance may have had no effect as people approaching this age were already using prescription drugs for chronic conditions. All except one [27] of the studies that examined the impact of user charges on patients' out-of-pocket expenditure on prescription drugs found that user charges increased patients' costs, while having additional voluntary health insurance coverage lowered their costs (see Table 5).

**Table 5:** The impact of prescription drug charges on patients' out-of-pocket expenditure on prescription drugs

**Variable**	**Out-of-pocket expenses**	**Studies**
Co-payment	+	**Stuart and Zacker **[123] (US, OS, CD, R)
Co-payment	0	**Lurk et al. **[27] (US, NS, CD, R)
Multi-tier formulary (vs. 1- or 2-tiers)	+	**Huskamp et al. **[124] (US, NS, CD, R); **Huskamp et al. **[125] (US, NS, CD, R); **Kamal-Bahl and Briesacher **[91] (US, OS, CD, R)
Reference pricing	+	**Grootendorst et al. **[101] (CA, NS, TD, R)
Insurance coverage		
Supplementary (vs. none)	-	**Alan et al. **[69] (CA, OS, CD, R); **Alan et al. **[68] (CA, OS, CD, R); **Alan et al. **[126] (CA, OS, CD, R); **Blustein **[127] (US, OS, CD, R); **Federman et al. **[114] (US, OS, CD, R)
Reimbursement limit	+	**Tseng et al. **[128] (US, OS, CD, NR)

Country: CA = Canada; US = United States

Type of study: ES = experimental study; NS = natural study; OS = observational study

Type of statistical analysis used: R = regression techniques; NR = no regression techniques

Thus, there is some evidence to suggest that cost sharing leads to slightly lower total expenditure or lower expenditure growth on prescription drugs and higher out-of-pocket expenditure for patients. The finding that patients are relatively insensitive to changes in the price of prescription drugs has important implications. Few of the studies we reviewed were of sufficient duration to permit assessment of long-term expenditure control. However, the reference price studies that took a slightly longer perspective found that reference pricing had little effect on expenditure beyond the first year or two, which suggests that user charges may not be relied upon to reduce pharmaceutical budgets in the long term. It also indicates that, rather than substantially lowering total expenditure on prescription drugs, user charges shift some prescription drug costs from third party payers to patients.

### 4.2. How do prescription drug charges affect total health care expenditure?

If user charges lower total expenditure on prescription drugs, they might also lower total expenditure on health care. Conversely, they could lead to a squeezed balloon effect, causing expenditure to rise in other parts of the pharmaceutical sector or health system. To assess the impact of prescription drug charges on total health care expenditure, we consider 23 papers that examined the relationship between prescription drug charges and the use of other forms of health care (a proxy indicator for expenditure) such as over the counter (OTC) drugs, doctor and outpatient visits, and inpatient and emergency care (see Table 6).

**Table 6:** Prescription drug charges and the demand for other health services

**Good/service affected**	**Variable**	**Effect**	**Study**
OTC drugs	Co-insurance	-	**Liebowitz **[32] (US, ES, CD, R)
	Insurance coverage		
	Supplementary (vs. none)	+	**Caussat and Glaude **[34] (FR, OS, CD, R)
	Supplementary (vs. none)	-	**Stuart and Grana **[33] (US, OS, CD, R)
	Prescription limit	+	**Cox et al. **[35] (US, OS, CD, R)
physician services	Co-payment	-	**Anis et al. **[129] (CA, OS, PD, R); **Balkrishnan et al. **[130] (US, NS, PD, R); **Lauterbach et al. **[131] (GE, OS, CD, R); **Winkelmann **[132] (GE, NS, PD, R); **Winkelmann **[133] (GE, NS, CD, R)
	Co-payment	0	**Gardner et al. **[29] (US, NS, TD, R)
	Multi-tier formulary (vs. 1- or 2-tiers)	0	**Motheral and Fairman **[28] (US, NS, CD, R)
	Reference pricing	-	**Hazlet and Blough **[36] (CA, NS, TD, R)
	Mixed system	+	**Li et al. **[31] (CA, NS, PD, R)
	Change from		
	co-insurance to deductible and co-insurance	0	**Pilote et al. **[30] (CA, NS, CD, R)
	Insurance coverage		
	Public Supplementary (vs. private)	+	**Raynaud **[134] (FR, OS, CD, R)
	Reimbursement limit	-	**Hsu et al. **[135] (US, OS, PD, R)
outpatient services	Co-payment	+	**Balkrishnan et al. **[130] (US, NS, PD, R)
	Mixed system	+	**Gaynor et al. **[99] (US, OS, PD, R)
	Insurance coverage		
	Public supplementary (vs. none)	+	**Raynaud **[134] (FR, OS, CD, R)
inpatient services	Co-payment	+	**Anis et al. **[129] (CA, OS, PD, R); **Atella et al. **[76] (IT, NS, CD, R); **Balkrishnan et al. **[130] (US, NS, PD, R)
	Co-payment	0	**Gardner et al. **[29] (US, NS, TD, R)
	Reference pricing	0	**Hazlet and Blough **[36] (CA, NS, TD, R)
	Multi-tier formulary (vs. 1- or 2-tiers)	0	**Motheral and Fairman **[28] (US, NS, CD, R)
	Mixed system	0	**Gaynor et al. **[99] (US, OS, PD, R)
	Insurance coverage		
	Supplementary (vs. none)	-	**Schoen et al. **[74] (US, NS, CD, NR)
	Public supplementary drug (vs. private)	-	**Lingle et al. **[136] (US, OS, CD, R)
	Prescription limit	0	**Soumerai et al. **[38] (US, NS, TD, R)
	Prescription limit	+	**Soumerai et al. **[37] (US, NS, TD, R)
	Reimbursement limit	+	**Hsu et al. **[135] (US, OS, PD, R)
ER visits	Multi-tier formulary (vs. 1- or 2-tiers)	0	**Motheral and Fairman **[28] (US, NS, CD, R)
	Reference pricing	+	**Hazlet and Blough **[36] (CA, NS, TD, R)
	Change from		
	co-payment to co-insurance and annual maximum	+	**Tamblyn et al. **[72] (CA, NS, TD, R)
	co-insurance to deductible and co-insurance	0	**Pilote et al. **[30] (CA, NS, CD, R)
	Reimbursement limit	+	**Hsu et al. **[135] (US, OS, PD, R)
	Prescription limit	+	**Soumerai et al. **[37] (US, NS, TD, R)
emergency mental health services	Prescription limit	+	**Soumerai et al. **[38] (US, NS, TD, R)
nursing home admissions	Prescription limit	+	**Soumerai et al. **[38] (US, NS, TD, R)

Country: CA = Canada; FR = France; GE = Germany; IT = Italy; US = United States

Type of study: ES = experimental study; NS = natural study; OS = observational study

Type of data analyzed: CD = cross-sectional data; TD = time-series data; PD = panel data

Type of statistical analysis used: R = regression techniques; NR = no regression techniques

As the use of prescription drugs requires a doctor's prescription, in most cases we would expect prescription drug charges to result in lower use of doctors (to the extent that patients are sensitive to changes in price). Several studies found that user charges, reimbursement limits, and reference pricing did indeed lead to a reduction in doctor visits; three studies found that prescription drug charges had no effect on doctor visits; and one study found that they increased doctor visits. However, two of the three studies finding no relationship between prescription drug charges and doctor visits examined situations in which user charges were designed to encourage the use of lower-cost drugs through multi-tier formularies [28] or differential charges for generics and brand-name medications [29], rather than to lower the use of prescription drugs. In the third of the three studies, the insignificant effect on doctor visits may be explained by the fact that everyone in the study sample had experienced a heart attack, while those with lower incomes were afforded greater protection from prescription drug charges [30]. Consequently, this group was less likely to be sensitive to changes in price and perhaps more likely to see the doctor for reasons other than to obtain a prescription. The positive result came from a sample of older people in Canada with rheumatoid arthritis [31]. As health insurance in Canada fully covers doctor visits, it is not surprising that some patients would substitute physician care for prescription drugs.

Studies show mixed results for OTC drugs. In the RAND experiment, higher co-insurance rates lowered the probability of purchasing an OTC drug but, after controlling for this, cost sharing had no impact on OTC expenditure [32]. Additional insurance coverage led to higher use of prescription drugs compared to OTC drugs in another US study [33], but had the opposite effect in a French study [34]. The French result probably differed because additional health insurance in France covers more than just prescription drugs; and as doctors often recommend the use of OTC drugs, increased OTC drug use may have been prompted by increased doctor visits. Having a limit on the number of free prescriptions an individual is allowed per month (a policy most often associated with Medicaid in the United States) positively influenced the quantity of OTC drugs used [35].

The results for outpatient, inpatient, and emergency care are much more consistent. As expected, user charges designed to encourage patients to choose lower-cost drugs had no significant effect on the use of inpatient or emergency care [28,36]. Otherwise, with the exception of four studies, there was generally a positive relationship between cost sharing and outpatient, inpatient, and emergency care. Studies also found that prescription limits increased the frequency of partial hospitalisation [37] and nursing home admissions [38] and the use of emergency mental health services [37]. Two of the studies that found no effect were based on chronically ill patients [30,38]. Soumerai et al. [38] also suggest that their insignificant result for inpatient admissions might be due to the fact that the outcome variable they used (time to first hospital admission) would not highlight repeat hospital visits.

These findings reveal two things. First, prescription drug charges are unlikely to lower total health care expenditure and may in fact increase spending overall. Although a decline in the use of services that complement prescription drugs (doctor visits) may lead to cost savings, any savings are likely to be outweighed by increased use of the highly resource-intensive services that substitute for prescription drugs (inpatient, emergency and long-term care). Second, the design of a cost sharing policy can mitigate this potentially explosive effect on total health care expenditure. Policies that give patients incentives to switch to lower-cost drugs and policies that protect low-income groups may prevent inefficient patterns of health care use which, while more accessible to patients, are more costly to the health system.

### 4.3. Is lower prescription drug expenditure achieved through reductions in price or quantity?

In this section we return to the question of total prescription drug expenditure and consider whether expenditure reductions resulting from user charges are achieved through reductions in the price of prescription drugs or reductions in the quantity of prescription drugs consumed. From a policy perspective, lowering expenditure through reductions in price would be preferable because, as the previous sections have shown, reductions in quantity can have unwelcome consequences for total health care costs and for equity (unless user charges exclusively reduce the use of unnecessary or ineffective prescription drugs).

#### Reductions in price

The effect of user charges on price reductions can be direct if reference pricing or tiered formularies encourage manufacturers to lower pharmaceutical prices, or indirect if they encourage patients to consume lower-priced prescription drugs. Fifteen studies examined direct or indirect price reductions attributed to prescription drug charges. For example, a Canadian study found that when a private insurer introduced a maximum allowable cost drug plan (similar in effect to reference pricing) for proton pump inhibitors, the price of inhibitors that were more expensive than the reference price fell [39]. A German study found that reference pricing led manufacturers to lower the price of medications in several therapeutic categories, with the largest reductions for brand-name drugs [40]. Danzon and Liu [41] found similar results for Germany, New Zealand, and the Netherlands. However, savings achieved through price reductions under reference pricing may be limited because manufacturers generally have no incentive to price products below the reference price. Savings may also be offset if, as has been observed, manufacturers respond to reference pricing by increasing the price of drugs not included in the reference pricing scheme [10].

There is more evidence about the way in which patients respond to incentives to switch to cheaper prescription drugs (see Table 7). Three studies that considered the impact of reference pricing found that patients immediately switched from drugs priced above the reference price to drugs priced at the reference price [42-44]. A study of the impact of differential co-payments for different statins (cholesterol-lowering drugs), ranging from $0 to $52.51, also found that patients were more likely to choose the cheapest option [45]. However, other studies that examined incentives to switch from brand-name to generic drugs show mixed results. Where user charges vary according to patients' insurance coverage, higher charges for brand-name drugs were successful in increasing demand for generic drugs [46,47]. In contrast, the RAND experiment found that co-insurance had no effect on the use of generic drugs [48,49], perhaps because generic substitutes were both less prevalent and more expensive in the United States during the 1970s and 1980s than they are at present [50]. More recent studies examining the impact of multi-tier formularies also found little impact on the use of generic substitutes, although there was some increase in the use of other therapeutic substitutes in the preferred tier. One reason for this may be that the studies focused either on an increase in user charges associated with a formulary or on a change from a two-tier to a three-tier formulary. While price-sensitive patients respond to an initial change from a one-tier to a two-tier formulary by increasing their uptake of generic drugs, patients who are less sensitive to price may continue to use brand-name drugs [51]. Subsequent price differentials between generics and brand-name drugs have little effect on the less sensitive group, but may encourage switching from non-preferred to preferred brand-name drugs.

**Table 7:** The impact of prescription drug charges on the use of generic or reference-priced drugs

**Variable**	**Use of generics**	**Use of other substitutes**	**Studies**
Co-payment	N/A	+	**Esposito**^1 ^[45] (US, OS, CD, R)
Multi-tier formulary (vs. 1- or 2-tiers)	0	+	**Huskamp et al. **[125] (US, NS, CD, R); **Motheral and Fairman **[28] (US, NS, CD, R)
Multi-tier formulary (vs. 1- or 2-tiers)	0	N/A	**Gibson et al. **[90] (US, NS, PD, R); **Motheral and Henderson **[63] (US, NS, CD, R)
Multi-tier formulary (vs. 1- or 2-tiers)	N/A	+	**Kamal-Bahl and Briesacher **[91] (US, OS, CD, R); **Landsman et al. **[65] (US, NS, TD, R);
Co-insurance	0	N/A	**Liebowitz et al. **[48] (US, ES, CD, R); **Newhouse **[49] (US, ES, CD, R)
Mixed system	+	N/A	**Hong and Shepherd **[46] (US, OS, CD, NR); **Mortimer **[47] (US, OS, CD, R)
Reference pricing (non-RP drugs)	+	N/A	**Marshall et al. **[42] (CA, NS, TD, R); **McManus et al. **[43] (AU, NS, TD, R); **Narine et al. **[44] (CA, NS, TD, NR)
Reference pricing (non-RP drugs)	N/A	+	**Mabasa and Ma **[39] (CA, NS, CS, NR)

Country: AU = Australia; CA = Canada; US = United States

Type of study: ES = experimental study; NS = natural study; OS = observational study

Type of data analyzed: CD = cross-sectional data; TD = time-series data; PD = panel data

Type of statistical analysis used: R = regression techniques; NR = no regression techniques

Overall, there is some evidence to suggest that reference pricing might be effective in encouraging drug manufacturers to lower their prices to the reference price, perhaps leading to a one-off reduction in expenditure on drugs in the reference pricing scheme. However, these savings may be offset by increases in the prices of cheaper drugs in the reference pricing scheme (to match the reference price) and increases in the price of drugs outside the reference pricing scheme. Evidence of the impact of patient-targeted incentives is mixed and, again, suggests the potential for minor and one-off cost savings only.

#### Reductions in quantity

Quantity can be affected by a decrease in the probability of consuming prescription drugs and/or a decrease in the volume (number) of prescription drugs consumed.

#### Impact on probability of use

Twenty-four articles used individual- or household-level data to examine the effect of prescription drug charges or insurance coverage on the probability of using any prescription drugs (see Table 8). The co-payments studied ranged in price from $0–$3 to approximately $33, and co-insurance rates ranged from 0% to 95%. With the exception of a Danish article [52], the studies were unanimous in finding that individuals who faced prescription drug charges were less likely to use prescription drugs and that those with insurance coverage were more likely to use them. The single unexpected result may be related to the fact that the authors did not control for endogeneity (in this case, the greater likelihood of unhealthier patients purchasing additional private health insurance) [52]. Only one study compared prescription drug consumption among individuals facing a change in the level of user charges (from a CDN $237 deductible and a 40% co-insurance rate to an income-based deductible with 0% co-insurance above the deductible; families with an annual income below CDN $15,000 faced a lower deductible) [53]. The change did not lead to any increase in the probability of consumption among low-income children, but did lower the probability of consumption among higher-income children. The income-based deductible therefore increased the out-of-pocket burden for higher-income households, but still proved to be a deterrent to the use of prescription drugs among lower-income households.

**Table 8:** The impact of prescription drug charges on the probability of obtaining a prescription drug

**Variable**	**Probability of use**	**Studies**
Co-payment	-	**Esposito**^1 ^[45] (US, OS, CD, R); **Gardner et al. **[137] (NZ, OS, CD, NR); **Hillman et al. **[138] (US, OS, CD, R); **Stuart and Zacker **[123] (US, OS, CD, R); **Watt et al. **[139] (NZ, OS, CD, NR)
Co-insurance	-	**Lohr et al. **[140] (US, ES, CD, NR)
Deductible	-	**Blais et al. **[141] (CA, NS, TD, R)
Mixed system	-	**Goldman et al. **[142] (US, OS, CD, R); **Ozminkowski et al. **[143] (US, OS, CD, R); **Smart and Stabile **[100] (CA, NS, CD, R)
Change from:		
deductible and co-insurance to income-based deductible	-	**Kozyrskyj et al. **[53] (CA, NS, CD, R)
Insurance coverage		
Primary (vs. none)	+	**Smith and Garner **[111] (US, NS, CD, NR); **Thomas et al. **[144] (US, OS, CD, R)
Supplementary (vs. none)	+	**Adams et al. **[145] (US, OS, CD, R); **Blustein **[127] (US, OS, CD, R); **Caussat and Glaude **[34] (FR, OS, CD, R); **Coulson and Stuart **[146] (US, OS, CD, R); **Genier et al. **[147] (FR, OS, CD, R); **Grignon and Perronin **[121] (FR, NS, CD, R); **Raynaud **[148] (FR, OS, CD, R); **Raynaud **[134] (FR, OS, CD, R); **Rogowski et al. **[149] (US, OS, CD, R); **Stuart and Grana **[33] (US, OS, CD, R)
Supplementary (vs. none)	0	**Christiansen et al. **[52] (DK, OS, CD, R)
Supplementary public (vs. private)	+	**Raynaud **[148] (FR, OS, CD, R); **Raynaud **[134] (FR, OS, CD, R)

^1^This study examined the probability of using a specific statin compared to the probability of using other statins when there were differing co-payments for each statin.

Country: CA = Canada; DK = Denmark; FR = France; NZ = New Zealand; US = United States

Type of study: ES = experimental study; NS = natural study; OS = observational study

Type of data analyzed: CD = cross-sectional data; TD = time-series data; PD = panel data

Type of statistical analysis used: R = regression techniques; NR = no regression techniques

#### Impact on volume

The bulk of the literature (92 studies) focuses on the impact of user charges on the volume of prescription drugs used (see Table 9). The co-payments studied ranged from $0–$3 to the full price of the drug. Co-insurance rates ranged from 0% to 100%. Most studies found a negative relationship between the prescription drug charge and levels of prescription drug use, regardless of the form of user charge in place. In most cases, insurance coverage had a positive effect on the volume of prescription drugs used, whereas the existence of a limited list of prescription drugs qualifying for reimbursement had a negative effect [54,55].

**Table 9:** The impact of prescription drug charges on the volume of prescriptions obtained

**Variable**	**Volume**	**Studies**
Co-payment	-	**Anderson et al. **[59] (SW, NS, TD, R); **Anessi Pessina **[150] (IT, OS, TD, R); **Anis et al. **[129] (CA, OS, PD, R); **Balkrishnan et al. **[130] (US, NS, PD, R); **Begg **[151] (UK, OS, CD, NR); **Birch **[152] (UK, NS, CD, NR); **Brenna et al. **[153] (IT, NS, TD, R); **Brian and Gibbens **[67] (US, ES, CD, NR); **Cameron et al. **[154] (AU, CD, NS, R); **Delnoij et al. **[155] (NE, NS, CD, R); **Gardner et al. **[137] (NZ, OS, CD, NR); **Gardner et al. **[29] (US, NS, TD, R); **Hansen et al. **[156] (US, OS, CD, R); **Harris et al. **[157] (US, NS, TD, R); **Hughes and McGuire **[158] (UK, NS, TD, R); **Hux et al. **[159] (CA, NS, CD, R); **Johnson et al. **[160] (US, NS, CD, R); **Johnson et al. **[161] (US, NS, CD, R); **Lauterbach et al. **[131] (GE, OS, CD, R); **Lavers **[162] (UK, NS, TD, R); **Livingstone et al. **[163] (CA, NS, CD, NR); **Lundberg et al. **[164] (SW, OS, CD, R); **Lurk et al. **[27] (US, NS, CD, R); **McManus et al. **[165] (AU, NS, TD, R); **Nelson et al. **[166] (US, NS, TD, R); **O'Brien **[54] (UK, NS, TD, R); **Reeder and Nelson **[61] (US, NS, TD, R); **Ryan and Birch **[55] (UK, NS, TD, R); **Schneeweiss et al. **[167] (CA, NS, TD, R); **Scott et al. **[168] (US, NS, CD, NR); **Smith and Watson **[169] (UK, OS, CS, R); **Starmans et al. **[170] (NE, NS, TD, R); **Watt et al. **[139] (NZ, OS, CD, NR)
Co-payment	0	**Anderson et al. **[59] (SW, NS, TD, R); **Meissner et al. **[66] (US, OS, CD, NR); **Reeder and Nelson **[61] (US, NS, TD, R); **Soumerai et al. **[62] (US, NS, TD, R)
Multi-tier formulary (vs. 1- or 2-tiers)	0	**Motheral and Henderson **[63] (US, NS, CD, R)
Multi-tier formulary (vs. 1- or 2-tiers)	-	**Fairman et al. **[23] (US, NS, CD, R); **Gibson et al. **[90] (US, NS, PD, R); **Landsman et al. **[65] (US, NS, TD, R); **Motheral and Fairman **[28] (US, NS, CD, R); **Rector et al. **[171] (US, OS, CD, R)
Co-insurance	-	**Foxman et al. **[172] (US, ES, CD, R); **Johnson et al. **[160] (US, NS, CD, R); **Johnson et al. **[161] (US, NS, CD, R); **Liebowitz et al. **[48] (US, ES, CD, R); **Lohr et al. **[140] (US, ES, CD, NR); **Martin and McMillan **[173] (US, NS, TD, R); **Puig-Junoy **[174] (SP, OS, TD, R); **Steffensen et al. **[175] (DK, NS, CD, NR)
Deductible	-	**Blais et al. **[141] (CA, NS, TD, R); **Socialstyrelsen **[176] (SW, OS, CD, NR)
Mixed system	-	**Carrin and Van Dael **[177] (BE, OS, TD, R); **Gaynor et al. **[99] (US, OS, PD, R); **Grootendorst and Levine **[56] (CA, OS, CD, R); **Hong and Shepherd **[46] (US, OS, CD, NR); **Klick and Stratmann **[25] (US, OS, CD, R); **Li et al. **[31] (CA, NS, PD, R); **Van Vliet et al. **[178] (NE, OS, CD, R)
Mixed system	+	**Grootendorst and Levine **[56] (CA, OS, CD, R)
Mixed system	0	**Mott and Schommer **[179] (US, OS, CD, R); **Ong et al. **[60] (SW, NS, TD, R)
Reference pricing (overall)	0	**Grootendorst et al. **[64] (CA, NS, TD, R)
Reference pricing (overall)	-	**Mabasa and Ma **[39] (CA, NS, CS, NR); **Schneeweiss et al. **[106] (CA, NS, TD, R)
Reference pricing (non-RP drugs)	-	**Grootendorst et al. **[64] (CA, NS, TD, R); **Mabasa and Ma **[39] (CA, NS, CS, NR); **Marshall et al. **[42] (CA, NS, TD, R); **McManus et al. **[43] (AU, NS, TD, R); **Narine et al. **[44](CA, NS, TD, NR)
Change from		
co-payment to co-insurance	-	**Van Doorslaer **[180] (BE, NS, TD, R)
co-payment to co-insurance and annual maximum	-	**Tamblyn et al. **[72] (CA, NS, TD, R)
co-insurance to deductible	-	**Friis et al**. [181] (DK, NS, CD, NR)
co-insurance to deductible and co-insurance	-	**Blais et al. **[182] (CA, NS, TD, R)
co-insurance to deductible and co-insurance	0	**Blais et al. **[57] (CA, NS, TD, R); **Pilote et al. **[30] (CA, NS, CD, R)
Insurance coverage		
Primary (vs. none)	+	**Artz et al. **[108] (US, OS, CD, R); **Coulson and Stuart **[146] (US, OS, CD, R); **Danzon and Pauly **[109] (US, OS, CD, NR); **Fillenbaum et al. **[183] (US, OS, CD, R); **Gianfrancesco et al. **[110] (US, NS, CD, NR); **Grootendorst et al. **[184] (CA, OS, CD, R); **Shih **[185] (US, OS, CD, R); **Smith and Garner **[111] (US, NS, CD, NR)
Primary (vs. none)	0	**Stuart et al **[58] (US, OS, CD, R)
Supplementary (vs. none)	+	**Caussat and Glaude **[34] (FR, OS, CD, R); **Coulson and Stuart **[146] (US, OS, CD, R); **Coulson et al. **[186] (US, OS, CD, R); **Davis et al. **[112] (US, OS, CD, NR); **Fillenbaum et al. **[183] (US, OS, CD, R); **Greenlick and Darsky **[187] (CA, OS, CD, NR); **Grootendorst et al. **[184] (CA, OS, CD, R); **Poisal and Chulis **[188] (US, OS, CD, NR); **Poisal and Murray **[117] (US, OS, CD, NR); **Rudholm **[189] (SW, OS, CD, R); **Stuart et al. **[119] (US, OS, CD, R); **Weeks **[120] (US, NS, CD, NR)
Limited list	-	**O'Brien **[54] (UK, NS, TD, R); **Ryan and Birch **[55] (UK, NS, TD, R)
Prescription limit	-	**Soumerai et al. **[62] (US, NS, TD, R); **Soumerai et al. **[37] (US, NS, TD, R)
Reimbursement limit	-	**Hsu et al. **[135] (US, OS, PD, R)

Country: AU = Australia; BE = Belgium; CA = Canada; DK = Denmark; FR = France; GE = Germany; IT = Italy; NE = The Netherlands; NZ = New Zealand; SP = Spain; SW = Sweden; UK = United Kingdom; US = United States

Type of study: ES = experimental study; NS = natural study; OS = observational study

Type of data analyzed: CD = cross-sectional data; TD = time-series data; PD = panel data

Type of statistical analysis used: R = regression techniques; NR = no regression techniques

Only 12 studies deviated from these intuitive findings to show either a positive correlation between out-of-pocket prescription drug price and volume [56] or no significant effect. In some cases this was due to study design. For example, studies found that cost sharing had a positive or no effect on the use of prescription drugs among groups of older people [56,57], among nursing home residents [58], among patients with chronic conditions [30,59,60], where user charges were low [59-62], or when patients were able to switch to cheaper alternatives. Older people are more likely to suffer from life-threatening and/or chronic conditions and may therefore be less sensitive to price. They may also perceive fewer substitutes for prescription drugs and, in some cases, decisions about drug use may be made by carers rather than the patients themselves [58]. The introduction of multi-tier formularies and reference pricing may negatively affect the volume of drugs that become relatively more expensive, but generally have little impact on overall volume as patients switch to less expensive drugs rather than curbing their consumption [63,64].

Several studies calculated estimates of the elasticity of demand for prescription drugs (or provided enough information for us to calculate estimates), with the aim of measuring the extent of patients' responsiveness to changes in the out-of-pocket price of prescription drugs (see Table 10). Overall, the demand for prescription drugs was almost always inelastic (less than proportionate). Studies generally found that a 10% increase in price would result in a 0.2 to 5.6% decrease in use based on non-aggregate data or a 0.6 to 8.0% decrease in use based on aggregate data. Estimates based on aggregate data are slightly larger due to higher levels of 'noise'. One study found an elastic decrease in use of 11.5% for tricyclic antidepressants, but inelastic decreases of 1.0 to 6.0% for other types of drug [65]. The evidence indicates that while patients are more sensitive to the price of brand-name drugs than to the price of generic drugs, the price elasticity of demand for the former is still relatively inelastic [47,63]. The Canadian study based on a sample of older people (see the previous paragraph) calculated a positive price elasticity estimate, suggesting that a 10% increase in price would actually lead to a 1.4% increase in use [56]. A further explanation for this may be that doctors attempted to ease the burden of higher prescription drug charges by increasing the size of prescriptions or prescribing cheaper drugs. The only other positive estimate came from a study that did not control for other factors that may have influenced demand, which may have biased the authors' calculation [66].

**Table 10:** Estimates of the elasticity of demand for prescription drugs^a^

**Paper**	**Type of cost sharing**	**Price elasticity**
**Anessi Pessina **[150] (IT, OS, TD, R)	Co-payment	-0.75 to -0.07
**Carrin and Van Dael **[177] (BE, OS, TS, R)	Mixed system	-0.35 to --0.09
**Coulson and Stuart **[146] (US, OS, CD, R)	Primary insurance (vs. none)	--0.18^b^
**Gardner et al. **[29] (US, NS, TD, R)	Co-payment	-0.38 to --0.23
**Gibson et al. **[90] (US, NS, PD, R)	Multi-tier formulary (vs. 1- or 2-tiers)	-0.27 to --0.03
**Grootendorst and Levine **[56] (CA, OS, CD, R)	Mixed system	-0.40 to 0.14
**Grootendorst et al. **[184] (CA, OS, CD, R)	Supplementary insurance (vs. none)	-0.13^b ^to --0.09^b^
**Harris et al. **[157] (US, NS, TD, R)	Co-payment	-0.17^b ^to --0.06^b^
**Hughes and McGuire **[158] (UK, NS, TD, R)	Co-payment	-0.37 to -0.32
**Klick and Stratmann **[25] (US, OS, CD, R)	Mixed system	-0.56
**Landsman et al. **[65] (US, NS, TD, R)	Multi-tier formulary (vs. 1- or 2-tiers)	-1.15 to -0.10
**Lavers **[162] (UK, NS, TD, R)	Co-payment	-0.22
**Li et al. **[31] (CA, NS, PD, R)	Mixed system	-0.20 to --0.11
**Liebowitz et al. **[48] (US, ES, CD, R)	Co-insurance	-0.10^b^
**McManus et al. **[165] (AU, NS, TD, R)	Co-payment	-0.80^b ^to --0.50^b^
**Meissner et al. **[66] (US, OS, CD, NR)	Multi-tier formulary (increase in co-payment for all tiers)	-0.22 to 0.39
**Mortimer **[47] (US, OS, CD, R)	Mixed system	-1.91 to --0.03
**Motheral and Henderson **[63] (US, NS, CD, R)	Multi-tier formulary (vs. 1- or 2-tiers)	-0.32^d^
**O'Brien **[54] (UK, NS, TD, R)	Co-payment	--0.64 to --0.23
**Puig-Junoy **[174] (SP, OS, TD, R)	Co-insurance	-0.13
**Ryan and Birch **[55] (UK, NS, TD, R)	Co-payment	-0.11 to --0.09
**Smith **[89] (US, OS, CD, R)	Mixed system	-0.10
**Smith and Watson **[169] (UK, OS, CD, R)	Co-payment	-0.58^c^
**Van Doorslaer **[180] (BE, NS, TD, R)	Change from co-payment to co-insurance	-0.60 to --0.06
**Van Vliet et al. **[178] (NE, OS, CD, R)	Deductible	-0.02

^a ^In some cases the authors of a paper may have reported a different kind of elasticity and where possible we have recalculated their estimates to reflect the standard definitions of elasticity. Whether we used an arc, point or constant elasticity calculation depended on the type of statistical analysis used and the kind of information reported by the authors.

^b^Calculated by the authors of this paper using the arc elasticity formula: *e*_*d *_= ((*Q*_2 _- *Q*_1_)/(*Q*_2 _+ *Q*_1_))((*P*_2 _+ *P*_1_)/(*P*_2 _- *P*_1_)).

^c^Calculated by the authors of this paper using a log-linear calculation: *e*_*d *_= *B*_*j*_$\bar{x}$, where *B*_*j *_represents the coefficient on the price variable and $\bar{x}$ is the mean price.

^d^Calculated by the authors of this paper using the point elasticity formula: *e*_*d *_= ((*Q*_2 _- *Q*_1_)/(*Q*_1_))((*P*_1_)/(*P*_2 _- *P*_1_))

Country: AU = Australia; BE = Belgium; CA = Canada; IT = Italy; NE = The Netherlands; SP = Spain; UK = United Kingdom; US = United States

Type of study: ES = experimental study; NS = natural study; OS = observational study

Type of data analyzed: CD = cross-sectional data; TD = time-series data; PD = panel data

Type of statistical analysis used: R = regression techniques; NR = no regression techniques

The evidence presented in this section shows that user charges reduce the use of prescription drugs, but not by much. In general, patients are relatively insensitive to changes in the out-of-pocket price of prescription drugs. The following sections focus on the sort of prescription drugs patients are most likely to forego and the type of patients most likely to respond to prescription drug charges.

### 4.4. What sort of prescription drugs are patients most likely to forego?

Policy makers often assume that patients will forego the use of drugs they value the least first and are therefore more likely to forego the use of non-essential than essential prescription drugs. Studies that considered this question generally defined essential drugs as those primarily used in the management of chronic conditions in which the cessation of drug therapy would have potentially serious consequences or as drugs that prevent deterioration in health or prolong life. Most studies found that prescription drug charges lowered the use of essential and non-essential drugs (see Table 11), although reductions in the use of non-essential drugs were usually slightly larger. This suggests that patients may attempt to discriminate on the basis of the usefulness of the prescription drug in question but are not always able to judge appropriately. Only one study found that cost sharing had little impact on the use of drugs classified as important for the treatment of serious illnesses or critical or necessary [67]. However, the study only reported descriptive statistics, and the small effect may have been due to the low level of the co-payment involved ($0.50 in 1971 dollars for the first two prescriptions in a month).

**Table 11:** The impact of prescription drug charges on the use of essential and non-essential medicines

**Variable**	**Use of essential medicines**	**Use of non-essential medicines**	**Studies**
Co-payment	-	-	**McManus et al. **[165] (AU, NS, TD, R)
Co-payment	0		**Brian and Gibbens **[67] (US, ES, CD, NR)
Co-insurance	-	-	**Foxman et al. **[172] (US, ES, CD, R)
Change from			
co-payment to co-insurance and annual maximum	-		**Tamblyn et al. **[72] (CA, NS, TD, R)
Prescription limit	-		**Fortress et al. **[190] (US, NS, PD, R); **Martin and McMillan **[173] (US, NS, TD, R); **Soumerai et al. **[62] (US, NS, TD, R); **Soumerai et al. **[38] (US, NS, TD, R)
Prescription limit		-	**Soumerai et al. **[62] (US, NS, TD, R)

Country: AU = Australia; CA = Canada; US = United States

Type of study: ES = experimental study; NS = natural study; OS = observational study

Type of data analyzed: CD = cross-sectional data; TD = time-series data; PD = panel data

Type of statistical analysis used: R = regression techniques; NR = no regression techniques

The finding that cost sharing reduces the use of essential as well as non-essential drugs suggests that, if policy makers are concerned about the impact of user charges on health (see below), they should not rely on patients to make the 'right' decisions about which drugs they may or may not forego.

### 4.5. Which patients are most likely to reduce their use of prescription drugs?

Prescription drug charges may adversely affect some groups of people more than others (for example, poorer people and heavy users of prescription drugs). Very few studies provide direct comparisons of reductions in prescription drug use among different population groups. However, Canadian researchers found that the introduction of additional insurance coverage lowered the proportion of a patient's budget spent on prescription drugs for older households with low and high levels of out-of-pocket spending on prescription drugs [68]. A later study confirmed this result and also found that among non-elderly households, the additional insurance coverage lowered out-of-pocket spending on prescription drugs more for low-income households than for high-income households [69]. This suggests that poorer households were most affected financially by user charges. At least in this context, it also indicates that increased user charges shifted expenditure from the government to poorer households. There was another interesting finding relating to specific types of insurance coverage. Although we might expect older Medicaid beneficiaries to spend more on prescription drugs than others due to the correlation between age, income, and health [70], poorer Medicare beneficiaries who were also eligible for Medicaid actually had lower prescription drug expenditure than those with Medicare only [71] (Medicare is the public insurance program for those aged 65 and above in the United States). This may be due to cost containment measures imposed by Medicaid, such as prescription restrictions and the use of formularies. A study that specifically addressed differences in essential drug consumption by income found that welfare recipients experienced greater reductions in essential drug use than older people, even when there was an annual out-of-pocket maximum in place [72].

Other studies indirectly compared sensitivity to prescription drug charges among different groups by comparing price elasticity estimates. Among the general population, price elasticity estimates showed that a 10% increase in price would lead to a 0.2 to 4.6% decrease in use based on non-aggregate data and a 0.9 to 8.0% decrease in use based on aggregate data. Changes in use for older people ranged from a 5.6% reduction to a 0.9% increase based on non-aggregate data, and the only study using aggregate data found a reduction of 5.1% [62]. Meanwhile reductions in use for poorer people ranged from 0.3 to 2.0% based on non-aggregate data and 0.5 to 4.0% based on aggregate data. These estimates suggest that poorer and older people are not more sensitive to price, in contrast to earlier literature reviews which found that poorer people were most sensitive and older people least sensitive to price [2,5]. Our results may differ due to newer estimates or estimates not considered in the earlier reviews. However, new statistical techniques such as meta-regression analysis also indicate that large variations in elasticity estimates may be influenced by publication bias (where journals favour papers with significant elasticity values), study characteristics, and institutional settings [73]. It is therefore difficult to make inferences regarding the distributional consequences of user charges based on elasticity estimates alone.

The few studies that have directly compared changes in the use of prescription drugs (in response to user charges) among different age or income groups have generally found that poorer people are more likely to lower their use when faced with higher prices. But there is a need for more research in this area, as the indirect evidence provided by elasticity estimates produces different results.

### 4.6. How do prescription drug charges affect health?

Datasets of sufficient longitude for the analysis required to assess the impact of user charges on health are scarce. Consequently, there are few direct attempts to answer this question. The RAND experiment found that there were only small differences in adult and child health status between those receiving 'free' care and those subject to user charges. However, it was not able to measure the long-term health effects of reductions in use, and few of its studies specifically linked prescription drug charges to health status. With regard to prescription drugs, the experiment found that people with higher levels of education used more OTC drugs and spent a larger proportion of their drug budget on OTC products (Leibowitz 1989). If, as this suggests, poorer people face financial and other barriers to access to OTC drugs (a substitute for prescription drugs), prescription drug charges are likely to have a greater negative effect on their health status.

Some non-experimental studies have also tried to assess the health impact of prescription drug charges. A Canadian study examined the impact of a change from no co-payment for people receiving social assistance and a small co-payment (CDN $2) with a CDN $100 out-of-pocket maximum for older people to 25% co-insurance with an annual maximum prescription charge of CDN $200–$925, depending on income [72]. The authors found that higher prescription charges increased hospitalisation rates, nursing home admissions, and mortality associated with reductions in the use of essential drugs. However, a separate study of the same change in user charges found that cost sharing had no impact on mortality or readmissions for complications among a group of older people who had experienced a heart attack [30]. Another study considered a natural experiment in which low-income patients with cardiovascular disease who did not have prescription drug coverage were given free drugs [74]. The study found that average blood pressure declined among patients with hypertension and average LDL fell among patients receiving free lipid-lowering drugs. A study of older people who had restricted their use of prescription drugs due to cost found that they experienced greater declines in health status compared with people who had not restricted use for cost reasons [75]. An Italian study looked at the correlation between co-payments, adherence to treatment and health in a sample of hypertension patients [76]. It found that the abolition of co-payments lowered the mortality rate for low-adherent patients by 0.7 percentage points, but had no effect on the mortality rate for high-adherent patients.

Other studies have attempted to address health outcomes indirectly, examining the effect of prescription drug charges on the use of essential vs. non-essential drugs (see Section 3.4) and on adherence to treatment. The financial burden imposed by user charges may induce patients to adopt strategies that affect adherence to a particular treatment regime. For example, patients may cut pills in half or skip doses. Most of the 22 studies that focused on adherence found that patients were less likely to adhere to treatment when faced with a co-payment, even if the co-payment was relatively small [77] (see Table 12). However, patients who faced co-payments were more likely to adhere to treatment regimes for diabetes than patients who faced co-insurance rates [78]. This difference may be due to patients' uncertainty about how changes in the price of drugs will affect their out-of-pocket spending under co-insurance [78]. Patients with co-payments may also be able to obtain larger prescriptions to avoid higher out-of-pocket costs, an option unavailable to those with co-insurance.

**Table 12:** The impact of prescription drug charges on adherence to treatment

**Variable**	**Compliance**	**Study**
Co-payment	-	**Atella et al. **[76] (IT, NS, CD, R); **Gibson et al. **[191] (US, OS, CD, R); **Poirier et al. **[77] (CA, NS, TD, NR)
Co-payment	0	**Poirier et al. **[77] (CA, NS, TD, NR)
Multi-tier formulary (vs. 1- or 2-tiers)	-	**Landsman et al. **[65] (US, NS, TD, R); **Shrank et al. **[79] (US, OS, CD, R); **Taira et al. **[80] (US, OS, CD, R)
Co-insurance	-	**Reuveni et al. **[192] (IS, OS, CD, R)
Mixed system	-	**Ellis et al. **[193] (US, OS, CD, R); **Goldman et al. **[194] (US, OS, CD, R); **Mojtabai and Olfson **[195] (US, OS, CD, R); **Piette et al. **[196] (US, OS, CD, R)
Change from		
co-payment to deductible and co-insurance	0	**Pilote et al. **[30] (CA, NS, CD, R)
Has co-insurance (vs. has co-payment)	-	**Dor and Encinosa **[78] (US, OS, CD, R)
Insurance coverage		
Primary (vs. none)	+	**Kennedy and Erb **[197] (US, OS, CD, NR); **Piette et al. **[196] (US, OS, CD, R); **Thomas et al. **[144] (US, OS, CD, R)
Primary public (vs. private)	-	**Dodrill et al. **[198] (US, OS, CD, NR)
Supplementary (vs. none)	+	**Col et al. **[199] (US, OS, CD, R**); Safran et al. **[200] (US, OS, CD, R); **Schoen et al. **[74] (US, NS, CD, NR); **Steinman et al. **[201] (US, OS, CD, R)
Prescription limit	-	**Cox et al. **[35] (US, OS, CD, R); **Schulz et al. **[81] (US, NS, CD, NR)

Country: CA = Canada; IS = Israel; IT = Italy; US = United States

Type of study: ES = experimental study; NS = natural study; OS = observational study

Type of data analyzed: CD = cross-sectional data; TD = time-series data; PD = panel data

Type of statistical analysis used: R = regression techniques; NR = no regression techniques

Adherence was also problematic for patients with a multi-tier formulary (vs. a two-tier formulary) [65] and for those who purchased non-preferred drugs in a multi-tier formulary (as opposed to generic or preferred drugs) [79,80]. Having any type of insurance coverage increased adherence to treatment, while the existence of a limit on the number of reimbursable prescriptions had the opposite effect [35,81]. The only study to find no effect on adherence examined the impact of a change from a CDN $2 co-payment to an income-related deductible (CDN $0 to $350) and 25% co-insurance with an income-related annual maximum (CDN $200 – $750) among a group of people who had all had experienced heart attacks [30]. As this paper focused on patients who were chronically ill and had experienced an adverse event related to their illness, it is not surprising that they adhered to their treatment. However, the study did not measure patients' adherence to other medications unrelated to cardiovascular disease, and it is possible that these patients lowered their adherence to other medications in order to be able to pay for cardiovascular treatment.

Overall, most studies that directly or indirectly considered the impact of prescription drug charges on health concluded that they lowered or were likely to lower health status because they led patients to forego the use of essential drugs, reduced adherence to treatment, and increased the likelihood of needing more intensive care and of dying. In the RAND experiment, the health effects of user charges (for all health services, not just prescription drugs) were more pronounced among low-income groups, in spite of an income-related annual maximum ceiling on out-of-pocket expenditure. This suggests that protection mechanisms do not always protect high-risk households.

## 5. What impact do prescription drug charges have on efficiency and equity?

There is no doubt that user charges reduce the use of prescription drugs and, therefore, enhance allocative efficiency as defined by standard welfare economics. However, as we argued at the beginning of this article, the unrealistic assumptions that must accompany a normative understanding of allocative efficiency limit its relevance to health policy. In assessing the impact of prescription drug charges on efficiency, we prefer an interpretation of efficiency more commonly used to evaluate policy: one that focuses on improving health through the provision of effective health care. From this perspective, the cost, health, and distributional consequences of prescription drug charges can be seen to lower efficiency. In the following paragraphs we examine the policy implications of each of these consequences in turn.

### Implications for health care costs

Almost all the studies we review conclude that prescription charges reduce the use of prescription drugs. However, they also show that most patients are not particularly sensitive to changes in the out-of-pocket price of prescription drugs. Put in economic terms, the demand for prescription drugs is price inelastic. This result is not surprising when we consider the pivotal role of doctors in prescribing drugs, which must have some bearing on patients' views about the necessity of taking such drugs, even when the financial outlay involved may be high. It may also be linked to unmeasured factors, including patient strategies to limit costs, such as obtaining higher doses of the same medication or cutting pills in half. But the implications for policy are profound. Because patients' overall response to prescription drug charges is muted, these charges fail to achieve large or long-term reductions in total prescription drug expenditure at the same time as they succeed in increasing patients' out-of-pocket spending on prescription drugs. What this suggests is that one of the main effects of prescription drug charges is to shift costs from third party payers (public or private) to patients. Indeed, a policy change in the Canadian province of British Columbia that slightly lowered prescription drug user charges for low-income groups and substantially increased them for middle- and higher-income groups directly transferred around CDN $134 million in prescription drug costs from the provincial government budget to patients, leading to substantial cost savings for the public purse [82].

There is some evidence to suggest that patients and producers respond to policies that enable patients to choose lower-cost drugs (for example, generic substitutes for brand-name drugs in multi-tier formularies or drugs available at the reference price), which has some effect on total and out-of-pocket expenditure on prescription drugs. However, as with reductions in the quantity of drugs consumed, the cost savings generated by reductions in price are usually limited and are one-off events. Nevertheless, by giving patients access to cheaper drugs, reference pricing or tiered formularies seem to prevent patients from turning to more expensive forms of free health care (inpatient, emergency, or long-term care) as a way of avoiding prescription drug charges. In contrast, standard prescription drug charges lead to increased use of these resource-intensive services, which results in higher levels of total expenditure on health care and undermines efforts to improve efficiency in the delivery of health care. From this we conclude that prescription drug charges should not be relied on to contain prescription drug costs in the longer term, or the costs of health care more generally. We recommend that policy makers monitor the impact of prescription drug charges (and other user charges) on the use of alternative health services.

### Implications for health

Few studies examined the impact of prescription drug charges on health, perhaps due to the difficulty of obtaining the long-term data required to do so. In general, those that directly considered health effects concluded that prescription drug charges increased the likelihood of needing more intensive care and, ultimately, of dying. The handful of studies that found that prescription drug charges had no negative effect on health usually focused on the use of drugs for chronic conditions among chronically ill people, a group we might expect to be less likely to lower use. A larger number of studies examined health effects indirectly, looking at the impact of prescription drug charges on adherence to treatment and the use of essential and non-essential drugs as proxy indictors for health. Most studies found that user charges lowered adherence to treatment and reduced the use of essential and non-essential drugs, strongly suggesting a negative impact on health.

The finding about reduced use of essential drugs indicates that the information asymmetry inherent in so many aspects of health care also affects the use of prescription drugs. Consequently, unless charging policies are specifically designed to direct patients towards or away from the use of particular types of drugs, their effect is likely to be indiscriminate. And if user charges adversely affect health, it is hard to see how they can lead to efficiency gains. Finally, reductions in the use of essential drugs suggest that in some cases patients may be unwilling or unable to pay for essential drugs, raising questions about equity.

### Implications for equity

If a key effect of prescription drug charges is to shift the costs of prescription drugs from public to private sources of finance, the impact on equity in finance is highly likely to be negative. International evidence consistently demonstrates that user charges are a regressive form of health care finance, requiring the poor to pay more for health care as a proportion of their income than the rich [83,84]. And in OECD countries, where levels of public spending on health care are usually high as a proportion of total spending, user charges undermine the equity (and efficiency) gains achieved by pooling financial resources across groups of people and over time.

Prescription drug charges are also likely to lower equity in the use of health care. Although elasticity estimates did not show low-income groups to be more sensitive to price than others, studies found that poorer people reduced their use of prescription drugs even when co-payment levels were very low. Low-income groups may already face significant financial and non-financial barriers to accessing prescription drugs, some possibly related to other cost control mechanisms in place, as the lower levels of drug expenditure incurred by Medicaid beneficiaries (vs. the privately-insured) in the United States suggest. Similarly, while older people seem to be less sensitive to price, the financial burden they face may be substantial if they are heavy users of prescription drugs. In the absence of substantial research into inequity in the use of prescription drugs, the results of studies examining inequity in the use of other forms of health care may be instructive. For example, international research has found significant pro-rich inequity in the use of general practitioners in the United States and Mexico but not in other high-income countries [85]. As prescription drug charges are more prevalent than other user charges, we infer that inequity in the use of prescription drugs may be an issue in some countries. Horizontal inequity may also be an issue in countries where prescription drug charges for low-income and older people can vary significantly from region to region (for example, in Canada and the United States). Further research in this area might contribute to lowering inequity in the use of prescription drugs and other forms of health care.

Correlations between income, age, and health [70], combined with some evidence to show that poorer people are sensitive to price and that the health effects of user charges (for all health services, not just prescription drugs) are more pronounced among low-income groups, suggest that policy makers should focus on protecting poorer and older groups and heavy users of prescription drugs from the financial burden of user charges. In some countries protection mechanisms in the form of exemptions or reduced rates cover groups not considered to be particularly vulnerable, such as high-income older people. This may be motivated by dislike of the administrative costs and stigma associated with means testing, forcing policy makers to balance concerns for equity with concerns for administrative efficiency and political fall-out. In other countries voluntary insurance may be the predominant protection mechanism, but as it only protects those who are able to pay for it, its impact may be limited and it may even exacerbate inequalities in access.

The extent to which protection mechanisms are effective depends on the context, particularly the form and scope of the protection mechanisms involved. Only a few studies have specifically examined this issue. A Canadian study found that a change from a fixed deductible to an income-related deductible was not adequate to protect low-income people [53]. Conversely, the introduction of drug benefit programs in another part of the country did manage to ensure access for low-income groups [68,69]. Where protection mechanisms target groups of people, individuals who are just above the threshold for exemption from prescription charges may be particularly disadvantaged. Again, few studies have explicitly addressed this question. Most of the relevant research involves US data and shows that individuals who are just above the Medicaid threshold are more likely to be uninsured than higher-income people and may therefore face substantial access barriers.

### Policy options

The evidence we have reviewed suggests two main options for policy makers wanting to use prescription drug charges to improve efficiency without lowering equity. First, enable patients to opt for cheaper alternatives such as generic vs. brand-name drugs or drugs that are cost-effective. While the cost savings involved may be limited, these policies have the advantage of contributing to efficiency in health care delivery. Second, introduce mechanisms to protect poorer people and heavy users of prescription drugs. Although research in these areas is limited, we suggest that smarter cost sharing systems would be carefully designed to ensure that protection mechanisms reflect need, are consistently applied, and do not conflict with other health policy goals. For example, voluntary insurance only protects those who can afford it and counteracts efforts to moderate demand, while fine-tuning exemption schemes through greater use of targeting (usually by means testing) may enhance equity at the expense of administrative efficiency. As a result of expanded exemption schemes, the burden of paying for prescription drugs may fall on the working population, who in many cases already make a significant contribution to financing health care. This involves economic and political trade-offs and decisions should reflect an open debate about values and goals. Similarly, while tiered formularies generate some cost savings, they may incur both administrative and political costs, particularly if they aim to promote cost-effectiveness.

Perhaps the smartest strategy would be to target those who research, manufacture, prescribe, and dispense drugs. Improvements in medical technologies (including drugs) leading to wider use are generally acknowledged to be the main driver of health care expenditure [86]. This implies a need for more research on the relationship between prescription drug charges and the diffusion of new technology. It also suggests that, rather than targeting patients through user charges, policy makers should focus on the incentives facing pharmaceutical companies, physicians, and pharmacists. The political economy of health systems may not make this an attractive option, but it is these groups who bear much of the responsibility for making decisions about the availability, use, and cost of prescription drugs.

## Competing interests

The authors declare that they have no competing interests.

## Authors' contributions

The article was conceived by MCG and EM, and the first draft was written by MCG and ST. Subsequent versions were revised by MCG, ST, and EM. All authors read and approved the final manuscript.
